# Supplementary figures and images for: A dual controllability analysis of influenza virus-host protein-protein interaction networks for antiviral drug target discovery
Source: BMC Bioinformatics. 2019 Jun 3;20:297. doi: 10.1186/s12859-019-2917-z (PMC6545738; doi:10.1186/s12859-019-2917-z)

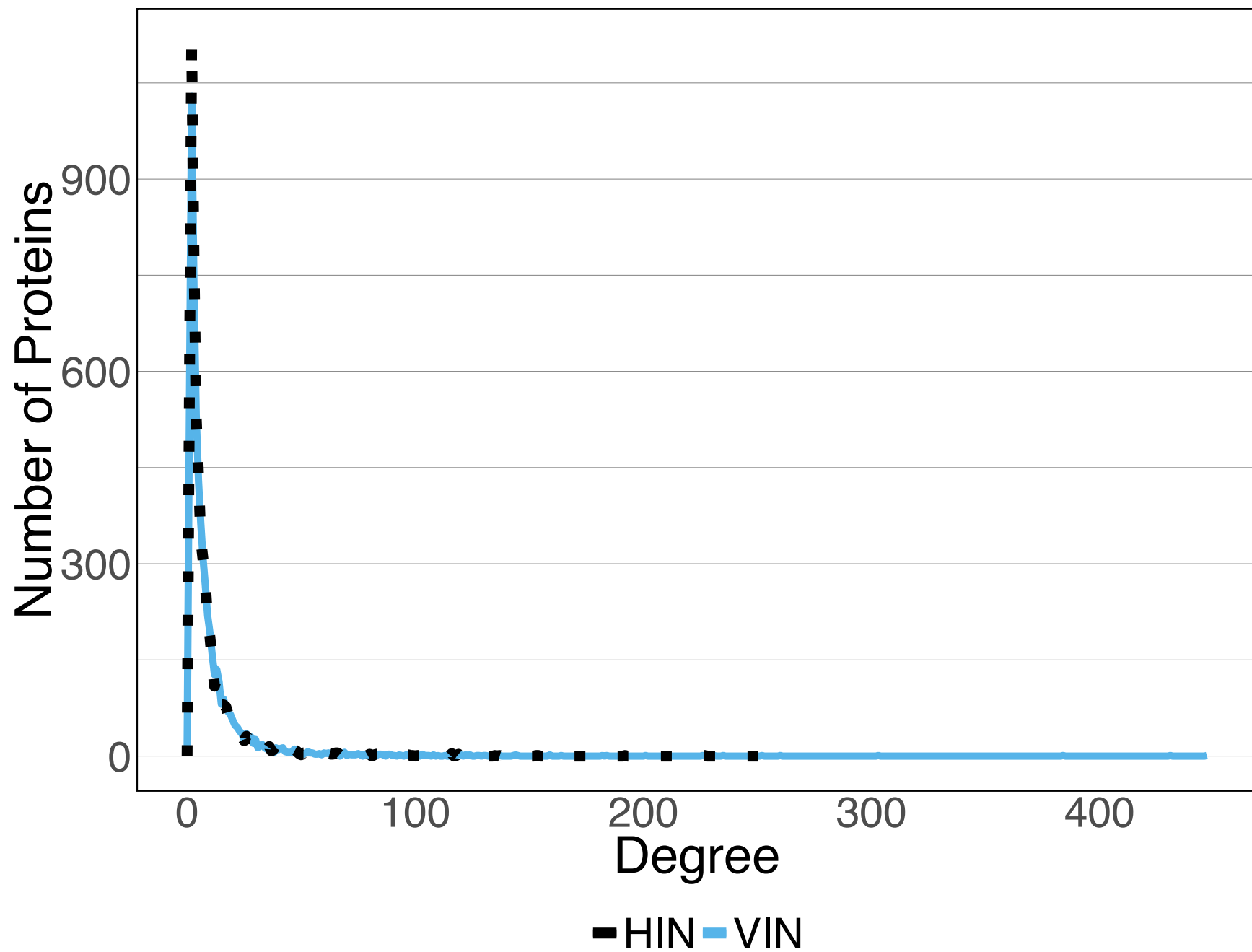

Supplement: Supplementary file 1 — Figure S1. Degree distribution of network with IAV interactions (blue solid) and without IAV interactions (dotted black) show that both networks demonstrate scale free topology (PDF 17 kb) [file 12859_2019_2917_MOESM1_ESM.pdf]
